# Fahr’s Syndrome Presenting With Hypocalcemia and Psychotic Features

**DOI:** 10.7759/cureus.18091

**Published:** 2021-09-19

**Authors:** Ahmed S Mohammedin, Abdullah F Alkharashi, Azzam A Alabdulqader, Hossain A Abualola, Mohammed A Serih

**Affiliations:** 1 Geriatric Medicine, Ain Shams University, Cairo, EGY; 2 Internal Medicine - Geriatrics, King Fahad Hospital of the University, Imam Abdulrahman Bin Faisal University, Khobar, SAU; 3 Radiology, King Fahad Hospital of the University, Imam Abdulrahman Bin Faisal University, Khobar, SAU

**Keywords:** epilepsy, brain calcification, seizures, hypoparathyroidism, bilateral striopallidodentate calcinosis, fahr's syndrome

## Abstract

Fahr's disease is a rare genetic neurodegenerative disorder described as “bilateral striopallidodentate calcinosis” (BSPDC). It is characterized by calcium deposition crossing the blood-brain barrier and calcifying different brain areas. Here, we report a case of a 26-year-old Saudi young lady, known as a case of epilepsy since childhood, a major depressive disorder with psychotic features, and hypocalcemia related to hypoparathyroidism. CT brain showed extensive coarse calcifications involving the infra and supratentorial white matter, predominantly within the basal ganglia, thalami, and dentate nuclei of cerebellar hemispheres. This report will discuss the challenging presentation, clinical symptoms, and the multidisciplinary approach to manage Fahr's syndrome symptoms. In conclusion, this case emphasizes the importance of neuroimaging and metabolic workup when investigating the seizure's etiology. The goal of treatment in Fahr's syndrome is to manage the underlying conditions.

## Introduction

Fahr's disease is a rare sporadic genetic neurodegenerative disorder described as bilateral striopallidodentate calcinosis (BSPDC), it is also labeled by another 35 different names in the previously reported cases [1]. It is characterized by calcium deposition crossing the blood-brain barrier and calcifying different brain areas, including basal ganglia, dentate nuclei, cerebral cortex, and other brain structures [1]. Various gene mutations were discovered associated with the disease, allowing a more precise disease character [2].

Fahr's disease has variable symptoms at presentation, including seizures, cognitive impairment, parkinsonism, tremor, dystonia, ataxia, chorea, dysarthria, headache, and other neuropsychiatric symptoms [3]. Here, we report a case of a 26-year-old Saudi female, with a past medical history of epilepsy since childhood, major depressive disorder with psychotic features, and hypocalcemia related to hypoparathyroidism associated with extensive coarse calcifications involving the infra and supratentorial white matter, predominantly within the basal ganglia, thalami, and dentate nuclei of cerebellar hemispheres. This report will discuss the challenging presentation, clinical symptoms, and the multidisciplinary approach to managing Fahr's syndrome symptoms.

## Case presentation

A 26-year-old young female presented with a history of partial seizures with secondary generalization since childhood. She was born to a non-consanguineous Saudi couple. The patient's seizures started at age of one month associated with decreased serum calcium levels. All history and lab were negative for other causes of seizures. She was diagnosed with partial seizure with secondary generalization and started phenobarbital at the age of one year, then tapered and discontinued at age of four years. During her childhood, she only had mild learning difficulties started at intermediate school, her school performance was challenging compared to her colleagues; however, she was able to finish high school and did not pursue higher education. Brain CT was normal initially. At the age of 21, she was diagnosed as having major depressive disorder with psychotic features and controlled on citalopram and quetiapine. At the age of 22, the patient presented to the emergency department (ER) complaining of dizziness after suddenly losing consciousness lasting about 30 seconds noticed by her mother; she had up rolling eyes with muscular rigidity. There was no urine incontinence, memory loss, or salivation, and she was seizure-free for the past three years until one month before this visit as when she again experienced one episode of seizure. Furthermore, the patient gave a history of multiple teeth loss throughout her life, and this description was suggestive of the manifestation of chronic hypocalcemia and dental enamel hypoplasia. Family history was negative for epilepsy, endocrine disorders, intellectual disability, and genetic diseases, she was the only case in her family. Also, the mother was not known to have any chronic medical illness, the pregnancy and antenatal history were unremarkable, and no genetic studies were done.

**Figure 1:**
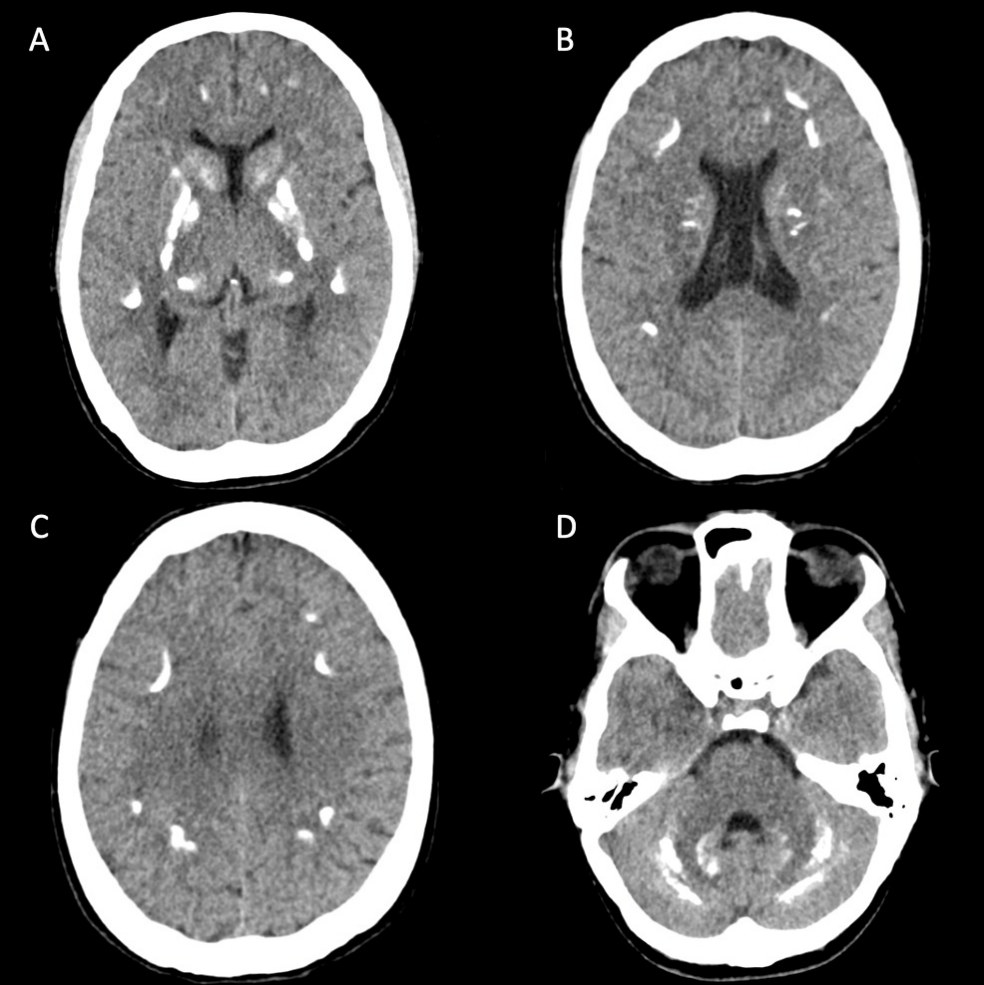
Brain CT without contrast (A) Axial nonenhanced head CT without contrast demonstrates fairly symmetric dense calcifications in the caudate, globus pallidi, putamen, and the thalami. (B, C) Images obtained at a higher level show multiple foci of dense calcifications in the centrum semiovale and at the gray-white matter junction. (D) Extensive diffuse hyperdense calcifications are also present throughout the cerebellum.

In ER, the patient was conscious, alert, oriented and communicating, stable vital signs, she had tiptoe walking during gait inspection, positive Chvostek's sign and teeth abnormalities. The patient was admitted to the medical ward for managing hypocalcemia. During the admission, the patient had total serum calcium level 5.1 mg/dL (normal range 8.5-10.1 mg/dL), serum phosphate: 6.2 mg/dL (normal range 2.6-4.7 mg/dL), magnesium level: 1.60 mg/dL (normal range 1.8-2.4 mg/dL), parathyroid hormone: 0.71 pmol/L (normal range 1.58-7.2 pmol/L), creatine phosphokinase: 777 U/L (normal range 26-308 U/L), thyroid-stimulating hormone: 1.127 µIU/mL (normal range 0.35-4.94 µIU/mL), total triiodothyronine level: 0.65 ng/mL (normal range 0.58-1.59 ng/mL), and total thyroxine level:4.24 mcg/dL (normal range 4.87-11.72 mcg/dL). Her ECG revealed prolonged QT interval, no echocardiography was done. A 21 channels EEG revealed an abnormal study showing a mild diffuse slowing of background rhythm and active focal epileptic discharges. These findings are suggestive of focal onset seizure with or without secondary generalization and right temporal structural pathology. Mild global cerebral dysfunction could be the result of underlying untreated epilepsy. A single brain CT without contrast was done has revealed: extensive coarse calcifications involving the infra and supratentorial white matter, predominantly within the basal ganglia, thalami, and dentate nuclei of cerebellar hemispheres (Figures 1A-1D). After receiving intravenous calcium gluconate and restoring the normal calcium level over 24 hours, she was discharged on calcium carbonate and alfacalcidol tablets.

Fahr's syndrome with primary brain calcification or secondary to treatment of hypoparathyroidism induced hypocalcemia was the provisional diagnosis; Based on her history, CT findings, and neuropsychiatric manifestation. Secondary causes of hypoparathyroidism were excluded, such as a history of thyroid surgery, congenital anomalies, history of neck radiation, Magnesium level was low during this visit, history of autoimmune disease, or history of malabsorption.

## Discussion

“Karl Theodor Fahr” reported in 1930 a case of 81 years old patient with dementia for a long time who presented complaining of high fever, cough, pressure ulcer, and immobility with no paralysis. After three days of the presentation, the patient died. Postmortem examination revealed calcification in striatum and centrum semiovale in addition to serous fluid filling the ventricles and granular cortex [4]. Interestingly, Fahr was not the first to describe the disease. The first person to report a case of bilateral calcification of basal ganglia was “A. Delacour” in the year 1850. He reported a case of 56 years old man who presented with lower limb weakness and stiffness plus tremor. The patient had severe diarrhea, hypotension, coma, bilateral vascular calcification, and basal ganglia stenosis after the postmortem pathological examination. As there was no history of such a case, he stated that further research was needed to understand the disease better [5]. Several cases were reported after Delacour with the same ambiguous disease. Due to the lack of terminology describing this neuro-mineral disease, more than 35 names were used to describe this disease in literature [1]. One commonly used name is Fahr disease. Pathological studies revealed the deposition of calcium and other minerals on the capillary and arterioles wall and the veins [6]. Moreover, the exact disease pathological processes are unknown; it was suggested that progressive inflammatory or metabolic processes within the brain lead to brain calcification, which results in neurological defects [7].

The disease could be classified depending on the etiology into two forms, i.e., primary and secondary [8]. The primary form "Fahr's disease" is familial, autosomal dominant, and sporadic. The secondary form "Fahr's syndrome" is the form that occurs in association with the presence of other underlying conditions. Regarding the clinical manifestation in symptomatic patients, movement disorder was the most common reported manifestation, followed by cognitive impairment, and speech disorder. Other reported manifestations are psychiatric features, pain, and sensory alteration [1]. Regarding the secondary causes of the disease, hypoparathyroidism is a metabolic cause and its treatment is considered the most common secondary cause [8]. Other secondary causes have been reported as toxoplasmosis, brucellosis, or acquired immune deficiency syndrome resulting in encephalitis [9]. Thirty percent of the systemic lupus erythematosus patients were found to have brain calcification with some clinical manifestation of Fahr syndrome [10]. Our patient fulfilled the diagnostic criteria of Fahr's syndrome [11-13]. She presented with neuropsychiatric features, which are progressive in nature, bilateral basal ganglia calcification, an association of hypoparathyroidism, and being young at the age of onset. There were no parkinsonism features in the case. Cognitive impairment could not be assessed as the case suffered from major depressive disorder with psychotic features and the family's main concern was epilepsy.

The modality of choice in diagnosing Fahr's syndrome is a non-contrast CT scan of the brain as it is the most sensitive radiological study [14]. Moreover, incidentally detecting intracranial calcification during the routine radiological study has been reported at a range of 0.3% to 1.2% in a prospective study [15]. The basal ganglia and cerebellum are the most common sites of calcifications [16]. Neurological and psychiatric symptoms vary depending on the calcification area in the brain, and this was discussed by Lopez-Vilegas et al. study when they reported 18 Fahr's syndrome patients with different neurological symptoms [17]. Furthermore, psychosis, mania, and different psychiatric conditions were found in 40% of the basal ganglia calcification patients [18]. Fahr's syndrome has no current cure to reverse calcification; it is only about treating the underlying conditions to the best of our knowledge. In our patient, she received antiepileptic drugs (phenobarbital then valproate), antidepressants (Citalopram), and antipsychotics (Quetiapine). She receives regular calcium replacement (calcium carbonate) and vitamin D for hypoparathyroidism. It is worth mentioning that calcium levels must be monitored and maintained as suppression of the seizure appears to be strongly associated with the normalization of calcium levels [19]. Whenever the diagnosis is made early, and the underlying condition is treated accordingly, the process of calcification could recede [9]. Recombinant parathyroid hormone was used as a line of management [20].

## Conclusions

In conclusion, Fahr's syndrome presents with a history of partial seizures with secondary generalization in addition to psychiatric manifestation. Upon investigations, primary hypoparathyroidism was diagnosed by laboratory workup, head CT of the patient revealed multiple calcifications predominantly in the basal ganglia bilaterally. Our case emphasizes the importance of neuroimaging and metabolic workup when investigating the etiology of the seizure. The goal of treatment in Fahr's syndrome is to manage the underlying conditions.
